# Testing the Efficacy of a Multi-Component DNA-Prime/DNA-Boost Vaccine against *Trypanosoma cruzi* Infection in Dogs

**DOI:** 10.1371/journal.pntd.0001050

**Published:** 2011-05-17

**Authors:** José E. Aparicio-Burgos, Laucel Ochoa-García, José Antonio Zepeda-Escobar, Shivali Gupta, Monisha Dhiman, José Simón Martínez, Roberto Montes de Oca-Jiménez, Margarita Val Arreola, Alberto Barbabosa-Pliego, Juan C. Vázquez-Chagoyán, Nisha Jain Garg

**Affiliations:** 1 Centro de Investigación y Estudios Avanzados, Universidad Autónoma de Estado de México, Toluca, México; 2 Department of Microbiology and Immunology, University of Texas Medical Branch, Galveston, Texas, United States of America; 3 Cardiology Clinic, Irapuato, México; 4 Department of Pathology, University of Texas Medical Branch, Galveston, Texas, United States of America; 5 Faculty of the Institute for Human Infections and Immunity, and the Sealy Center for Vaccine Development, University of Texas Medical Branch, Galveston, Texas, United States of America; René Rachou Research Center, Brazil

## Abstract

**Background:**

*Trypanosoma cruzi,* the etiologic agent of Chagas Disease, is a major vector borne health problem in Latin America and an emerging infectious disease in the United States.

**Methods:**

We tested the efficacy of a multi-component DNA-prime/DNA-boost vaccine (TcVac1) against experimental *T. cruzi* infection in a canine model. Dogs were immunized with antigen-encoding plasmids and cytokine adjuvants, and two weeks after the last immunization, challenged with *T. cruzi* trypomastigotes. We measured antibody responses by ELISA and haemagglutination assay, parasitemia and infectivity to triatomines by xenodiagnosis, and performed electrocardiography and histology to assess myocardial damage and tissue pathology.

**Results:**

Vaccination with TcVac1 elicited parasite-and antigen-specific IgM and IgG (IgG2>IgG1) responses. Upon challenge infection, TcVac1-vaccinated dogs, as compared to non-vaccinated controls dogs, responded to *T. cruzi* with a rapid expansion of antibody response, moderately enhanced CD8^+^ T cell proliferation and IFN-γ production, and suppression of phagocytes’ activity evidenced by decreased myeloperoxidase and nitrite levels. Subsequently, vaccinated dogs controlled the acute parasitemia by day 37 pi (44 dpi in non-vaccinated dogs), and exhibited a moderate decline in infectivity to triatomines. TcVac1-immunized dogs did not control the myocardial parasite burden and electrocardiographic and histopatholgic cardiac alterations that are the hallmarks of acute Chagas disease. During the chronic stage, TcVac1-vaccinated dogs exhibited a moderate decline in cardiac alterations determined by EKG and anatomo-/histo-pathological analysis while chronically-infected/non-vaccinated dogs continued to exhibit severe EKG alterations.

**Conclusions:**

Overall, these results demonstrated that TcVac1 provided a partial resistance to *T. cruzi* infection and Chagas disease, and provide an impetus to improve the vaccination strategy against Chagas disease.

## Author Summary

Immunization of dogs with DNA-prime/DNA-boost vaccine (TcVac1) enhanced the *Trypanosoma cruzi*-specific type 1 antibody and CD8^+^ T cell responses that resulted in an early control of acute parasitemia and a moderate decline in pathological symptoms during chronic phase. Further improvement of vaccine-induced immunity would be required to achieve clinical and epidemiological benefits and prevent transmission of parasites from vaccinated/infected dogs to triatomines.

## Introduction

American trypanosomiasis (Chagas disease) is a disease of humans and caused by the protozoan *Trypanosoma cruzi* of the family trypanosomatidae [1]. Approximately 30–40% of the infected patients develop a chronic debilitating illness of the cardiac system, characterized by clinically irreversible and progressive tissue destruction, and myocardial hypertrophy, eventually leading to heart failure and the patient’s death [2], [3].

Several investigators have shown the potential utility of *T. cruzi* surface antigens as vaccine candidates in murine experimental models [4], [5](reviewed in [6], [7]). We have shown the protective efficacy of amastigote surface proteins ASP-1 and ASP-2, and trypomastigote surface antigen TSA-1 as DNA vaccines in mice [8]. Vaccination with ASP-2 provided maximal immunity to *T. cruzi* infection in mice that was further enhanced by co-delivery of cytokine adjuvants [8]. In recent studies, we have identified additional potential vaccine candidates by computational screening of *T. cruzi* sequence database [9]. Of these, TcG1-TcG8 were phylogenetically conserved in clinically important strains of *T. cruzi* and expressed in the infective and intracellular stages of the parasite [9]. When delivered as a DNA vaccine in mice, TcG1, TcG2 and TcG4 elicited a significant trypanolytic antibody response and Th1 cytokine (IFN-γ) response, a property associated with immune control of *T. cruzi* [10]. These novel vaccine candidates, thus, increased the pool of protective vaccine candidates against *T. cruzi*.

In this study, we proceeded to examine the prophylactic and transmission-blocking efficacy of the multi-component vaccine constituted of TcG1, TcG2 and TcG4 in dogs. We chose dogs for our studies because dogs provide an excellent model for studying the human disease [11]–[13]. Experimentally and naturally infected young dogs (2–3 months) elicit reproducible and comparable acute infection associated with increase in blood parasitemia, IgG and IgM antibodies [14], [15] and T cell response [16]. The presence of myocarditis with a moderate or small number of parasitized cells and extensive and frequent focal necrosis turns the disease in dogs similar to acute Chagas disease in humans [17], [18]. A few infected dogs (10–20%; 5% humans) develop severe acute myocarditis and may die of cardiac arrest. More than 80% of dogs recover from acute parasitemia as parasites become undetectable in the blood, and remnant mild myocardial changes with scattered microscopic foci of fibrosis and lymphocytic infiltration [19] present a picture similar to that of human infections [20], [21]. At 12–18 months post-infection (pi), ∼50% of infected dogs exhibit symptomatic chronic cardiac disease (20% develop severe myocarditis), associated with progressive cardiomegaly; arrhythmia, including RBBB with left anterior hemiblock [22], [23]; diffused myocarditis with focal and interstitial fibrosis, and self-perpetuating myofibril destruction - a picture reminiscent of the chronic form of Chagas disease observed in humans. Thus, testing the vaccine efficacy in dogs would provide a strong basis for developing a human vaccine against *T. cruzi* and Chagas disease.

Further, dogs are an important reservoir host for domestic transmission of *T. cruzi*. The prevalence rate of *T. cruzi* infection in dogs may reach up to 84% in endemic areas (e.g. rural Argentina, Chiapas, Mexico), determined by serological procedures and xenodiagnosis [24], [25]. Dogs are also the most frequent blood meal source for the domestic triatomines, i.e., *T. barberi* and *T. pallidipennis* in Mexico [15] and *T. infestans* in Argentina [25], [26]. Likewise, a high prevalence of seropositive dogs and infected triatomines is routinely noted in rural and urban development in the southern US [27], [28], and suggested to maintain *T. cruzi* transmission in the human habitat. Triatomines are several times more likely to take their blood meal from dogs than from humans [26]. The ratio of dog blood meals to human blood meals in the engorged guts of triatomines is estimated to be 2.3–2.6 times higher than the ratio of the number of dogs to the number of humans in a household [29]. Thus, the probability of infecting an insect in one blood meal from dogs is estimated to be 200-times higher as compared to the probability from adult humans [25]. These studies demonstrate that dogs are an important host blood source for domiciliary triatomines, and the risk of *T. cruzi* infection in humans is increased by the presence of infected dogs. Strategies that can limit *T. cruzi* infection in domestic reservoir host may, thus, prove to be effective in interrupting the parasite transmission to the vector, and consequently, to the human host.

We immunized dogs with DNA-prime/DNA-boost vaccine (TcVac1). We examined the efficacy of TcVac1 in eliciting antigen-and parasite-specific antibody and T cell immunity, and determined if vaccination with TcVac1 modulated the host immune response towards protective type 1 upon *T. cruzi* infection. We also examined the efficacy of TcVac1 in controlling acute parasitemia, blocking the parasite transmission to triatomines, and preventing clinical severity of chronic disease.

## Materials and Methods

### Animals

Twelve mongrel dogs (6 males and 6 females, 3–4 months old) were acquired locally and kept at the animal facility at the UAEM Research Center until they were included in the experiment, at eight months of age (8–12 kg body weight). Dogs were confirmed free of *T. cruzi* infection by microscopic examination of blood smears and serological evaluation of anti-*T. cruzi* antibodies using an indirect haemagglutination assay (IHA) and enzyme-linked immunosorbent assay (ELISA) [15], [18]. Before inclusion in experimental studies, dogs were treated with anti-helminthes and vaccines against regional infectious diseases (Canine distemper, Parvovirus infection, Canine hepatitis, Leptospirosis, and Rabies). All dogs received water *ad libitum,* and commercial dog food fed twice a day according to their age and development requirements. Experimental protocols were conducted under the technical specifications for the production, care, and use of lab animals from the Norma Official Mexicana (NOM-062-ZOO-1999), and the Council for International Organizations of Medical Science [30], [31]. The research protocols were approved by the Laboratory Animal Care Committee at the Universidad Nacional Autonoma de Mexico.

### Immunization and challenge infection

TcVac1 vaccine was constituted of antigen-encoding plasmids (pCDNA3.TcG1, pCDNA3.TcG2 and pCDNA3.TcG4) and IL-12- and GMCSF-expression plasmids, described previously [8], [32]. The eukaryotic expression plasmids encoding dog cytokines (IL-12 and GM-CSF) were a kind gift from Dr. Peter Melby [33]. All recombinant plasmids were transformed into *E. coli* DH5-α competent cells, grown in L-broth containing 100 µg/ml ampicillin, and purified by anion exchange chromatography using the Qiagen maxi prep kit (Qiagen, Chatsworth, CA) according to the manufacturer’s specifications. Trypomastigotes of *T. cruzi* (SylvioX10/4) were maintained and propagated by continuous *in vitro* passage in C2C12 cells.

Dogs (n = 6/group, 3 males and 3 females) were intramuscularly immunized with TcVac1 (200 µg each plasmid DNA/dog), delivered four-times at 2-week intervals. Dogs vaccinated with empty vector (pcDNA3 only) were used as controls. Two-weeks after the last immunization, dogs were challenged with culture-derived *T. cruzi* SylvioX10/4 (3.5×10^3^ trypomastigotes/kg body weight, i.p.). The selected dose of the parasites was sufficient to produce acute parasitemia within 1–2 weeks of inoculation, and symptomatic clinical disease within 6–8 weeks post-infection [18]. Dogs were observed daily for general physical condition, at weekly intervals for clinical condition, and at 2-week intervals for cardiac function, monitored by electrocardiography (EKG). Sera samples were obtained before each immunization and at two-week intervals thereafter. After challenge infection, in addition to sera samples, blood samples for parasitemia diagnostics were collected beginning day 5 pi, on alternate days up to 50 dpi and at two-week intervals thereafter.

### Parasitological measures

We measured blood parasitemia using hemacytometer counts of 5 µl blood mixed with equal volume of ACK red blood cell lysis buffer. Xenodiagnostic analysis was performed as described [25], [34], [35]. Briefly, stage 4 naive triatomine (*T. pallidipenis*) nymphs (6 per dog) were fed on vaccinated and control dogs on day 30 and day 60 pi. Fecal samples were collected from triatomines at day 60 after feeding, and analyzed by light microscopy to detect epimastigote and metacyclic trypomastigotes. At least 10 microscopic fields were analyzed for each fecal sample, and triatomines were considered *T. cruzi* positive when >1 parasites were detected.

### Serology

The cDNAs for *TcG1, TcG2* and *TcG4* were cloned in pET-22b plasmid (Novagen) such that the encoded proteins would be expressed in-frame with a C-terminal His-tag. All cloned sequences were confirmed by restriction digestion and sequencing at the Recombinant DNA Core Facility at UTMB. For the purification of recombinant proteins, plasmids were transformed in BL21 (DE3) pLysS competent cells, and recombinant proteins purified using the polyhistidine fusion peptide-metal chelation chromatography system (Novagen).

Blood samples were obtained by venopuncture of the cephalic vein, and immediately processed to separate sera, using standard methods [15], [18]. Sera samples (1∶50–1∶100 dilution) were analyzed for IgM and IgG by using the Chagas diagnostic kits for ELISA (Laboratorio-Lemos SRL, Buenos Aires, Argentina). The horseradish peroxidase (HRP)-labeled anti-human-IgG in ELISA kit was replaced with HRP-conjugated goat-anti-dog IgM- or IgG-specific secondary antibody (Bethyl Laboratories) [15], [18]. In some experiments, instead of *T. cruzi* lysate, plates were coated with recombinant antigens (TcG1, TcG2 or TcG4, 10-µg protein/ml) to capture the antigen-specific antibodies. Sera samples from chronically infected dogs with confirmed *T. cruzi* infection and from healthy domestic dogs were used as positive and negative controls, respectively (cut off value: ELISA, mean OD_450nm_ from negative dogs ±2 SD; IHA, positive titer at ≥1∶16 serum dilution).

To identify the antibody sub-types (IgG1 and IgG2), plates were coated with *T. cruzi* antigen, and, then sequentially incubated at room temperature with sera samples (1∶50 dilution) for 2 h, biotin-conjugated goat anti-dog Ig subtypes (IgG1 and IgG2) for 2 h, and streptavidin-horseradish peroxidase conjugate for 30 min. All antibodies and conjugates were from Bethyl Laboratories, and used at a 1∶3000 dilution in PBST-0.5% NFDM (100-µl/well). Color was developed with 100-µl/well Sure Blue TMB substrate (Kirkegaard & Perry Labs), reaction was stopped with 2N sulfuric acid, and antibody response was monitored at 450 nm using a SpectraMax M5 microplate reader.

### Spleen cell phenotype

Splenic level of CD4^+^ and CD8^+^ cell population in immunized/challenged dogs was determined by flow cytometry. Briefly, splenocytes were suspended in PBS (1×10^6^ cells/100 µl) and incubated for 30 min with FITC-conjugated anti-CD4 and PE-conjugated anti-CD8 antibodies (1∶50 dilution, from ABD Serotec). Following incubation, cells were fixed with 2% paraformaldehyde, washed and re-suspended in 500 µl PBS, and analyzed on a FACScan apparatus (BD Biosciences). Cells stained with PE- and FITC- conjugated rat IgGs (isotype matched) were used as negative controls. Flow data were analyzed by Cell Quest software (BD Biosciences).

### Serum cytokine levels

Cytokine levels in sera of vaccinated dogs were measured by sandwich ELISA. Briefly, 96 well plates were coated overnight with anti-IFN-γ or anti-IL-10 antibodies (500-ng/ml in PBS), washed with PBS/0.05% Tween-20 (PBST), and incubated for 2 h with 1% BSA. Plates were then sequentially incubated at room temperature with sera samples (50-µl/well) for 2 h, biotinylated anti-dog IFN-γ antibody (0.5-µg/ml) or anti-dog IL-10 antibody (2-µg/ml) for 2 h, and streptavidin conjugated horse radish peroxidase (1∶3000 dilution) for 45 min. All antibodies were from R&D systems. Colorimetric reaction was performed as above. Cytokine concentrations were calculated using a standard curve derived using recombinant IFN-γ or IL-10 (1–4000 pg/ml).

### Myeloperoxidase (MPO) activity

MPO activity was determined by a dianisidine-H_2_O_2_ method [36], modified for 96-well plates [37]. Briefly, plasma samples (10-µg protein) were added in triplicate to 0.53 mM *o*-dianisidine dihydrochloride (Sigma) and 0.15 mM H_2_O_2_ in 50 mM potassium phosphate buffer (pH 6.0). After incubation for 5 min at room temperature, the reaction was stopped with 30% sodium azide and the change in absorbance was measured at 460 nm (ε = 11,300 M^−1^ cm^−1^). Results were expressed as units of MPO/mg protein, whereby one unit of MPO was defined as the amount of enzyme degrading one n mol H_2_O_2_ per min at 25°C.

### Nitrite level

The nitrite/nitrate content, indicative of inducible nitrite oxide synthase (iNOS) activity, was monitored by the Greiss reagent assay, as described [37]. In 96-well plates, reduced plasma samples (10 µg protein) were mixed with 100 µl Greiss reagent, consisting of 1% sulfanilamide in 5% phosphoric acid and 0.1% *N*-(1-napthyl)ethylenediamine dihydrochloride (1∶1, v/v), and incubated for 10 min. The change in absorbance was monitored at 545 nm (standard curve, 0–200 n mol sodium nitrite).

### Electrocardiography

Changes in cardiac rhythm and conduction in all dogs was monitored before inclusion in the study, and after challenge infection, at 2-week intervals up to 8-weeks and at monthly intervals thereafter. We used electrocardiograph (Stylus, EK-8, USA) setting at 120 V, 60 Hertz, 20 amps, and 25 Watt in all experiments. Six leads of the electrocardiogram were considered at 25 mm/sec at 1-mV, standardized to 1 cm for the present study.

### Necropsy and histological studies

Necropsy was performed the day animals died due to infection or after humanitarian sacrifice at day 60 (acute phase) and day 365 (chronic phase) post challenge infection. Dogs were sedated with xylazine (1–3 mg/kg body weight) and then euthanized according to the Mexican Norma Official Mexicana [30], [31], using protocols approved by the Laboratory Animal Care Committee at the Universidad Nacional Autonoma de Mexico. A macroscopic and microscopic analysis of affected organs was performed. Postmortem studies were conducted using standard protocols with emphasis on macroscopic findings related to Chagas disease in heart tissue [15]. For histological analysis, tissue samples were fixed in 10% buffered formalin for 24 h, dehydrated in absolute ethanol, cleared in xylene, and embedded in paraffin. Tissue sections (5-µm thick) were stained with hematoxylin-eosin, and evaluated by light microscopy at 100× and 400× [15], [18]. Tissues were scored 0 to 4 in blind studies, according to the extent of inflammation and tissue damage from normal to total wall involvement [38].

### Statistical analysis

Data are expressed as means ± SD, and derived from duplicate experiments (n≥6 animals/group/experiment) with at least duplicate observations per sample. Results were analyzed for significant differences using ANOVA procedures and Student’s t-tests. The level of significance was accepted at *p<0.05 (vaccinated versus non-vaccinated).

## Results

**Figure 1:**
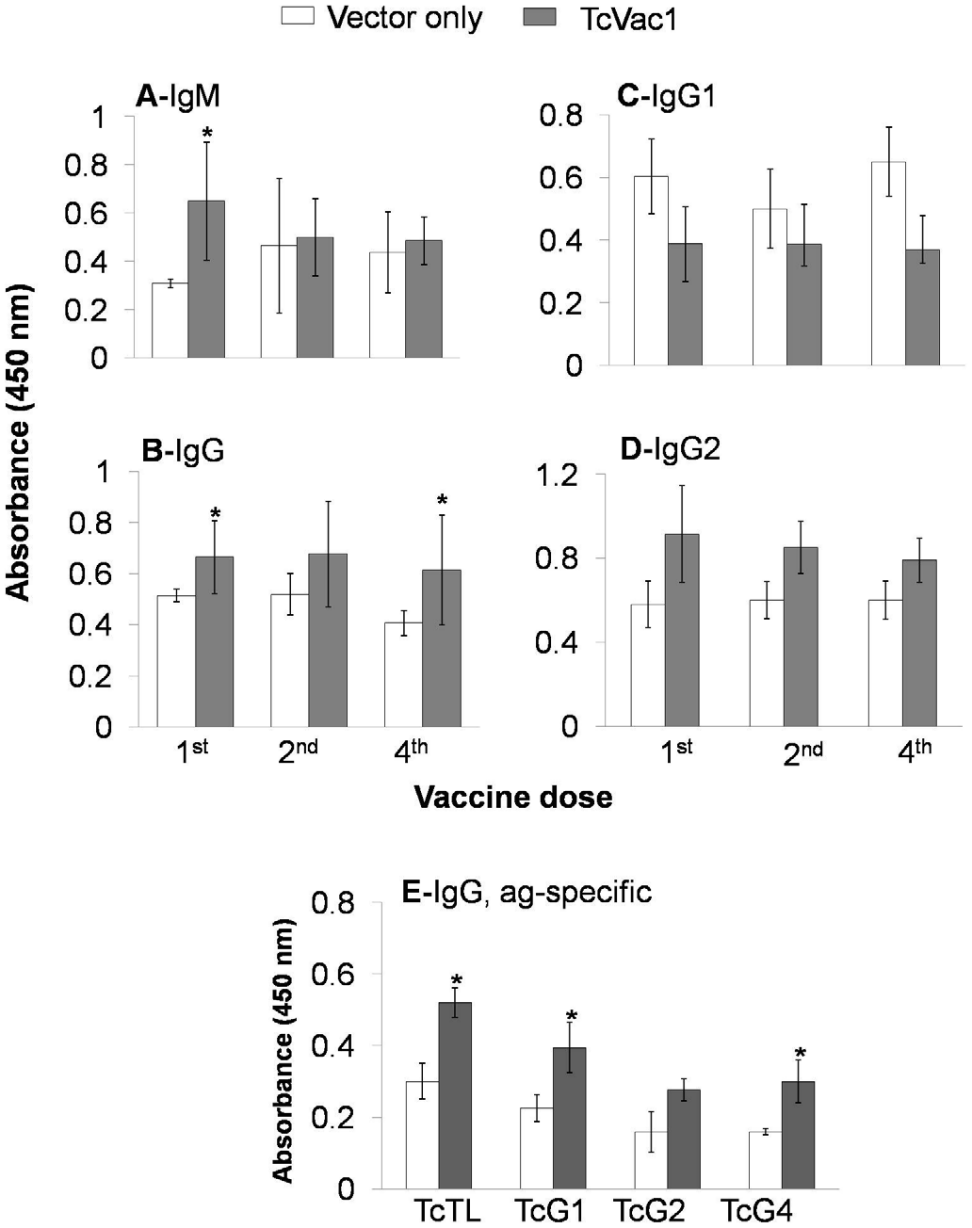
TcVac1-induced antibody response in dogs. Dogs were vaccinated with TcVac1 or injected with vector only, and sera were collected two weeks after each immunization dose. An ELISA was performed to monitor the sera levels (1∶50 dilution) of parasite-specific IgM (**A**), IgG (**B**), IgG1 (**C**) and IgG2 (**D**). The vaccine-induced antigen-specific (TcG1, TcG2 and TcG4) antibody response in sera collected 14 days after last dose of vaccine was determined using recombinant antigens. Data are presented as mean ± SD (*p<0.05, vaccinated versus non-vaccinated, n = 6 per experiment).

The development of an antibody response induced by TcVac1 was determined by an ELISA. All dogs were seronegative before vaccination was initiated. The *T. cruzi*-specific IgM and IgG antibody response was detectable in sera (1∶50 dilution) of vaccinated dogs after the first immunization, and moderately increased upon delivery of booster vaccine doses (Fig. 1A&B). The level of antigen-specific antibody response was detected in the order of TcG1>TcG2>TcG4, and was additive in nature (Fig. 1E). The vaccine-induced antibody response was predominantly of the Th1 type with IgG2/IgG1 ratios >1 (Fig. 1C&D). Control dogs immunized with plasmid vector alone exhibited no parasite- and antigen-specific antibody response (Fig. 1).

**Figure 2:**
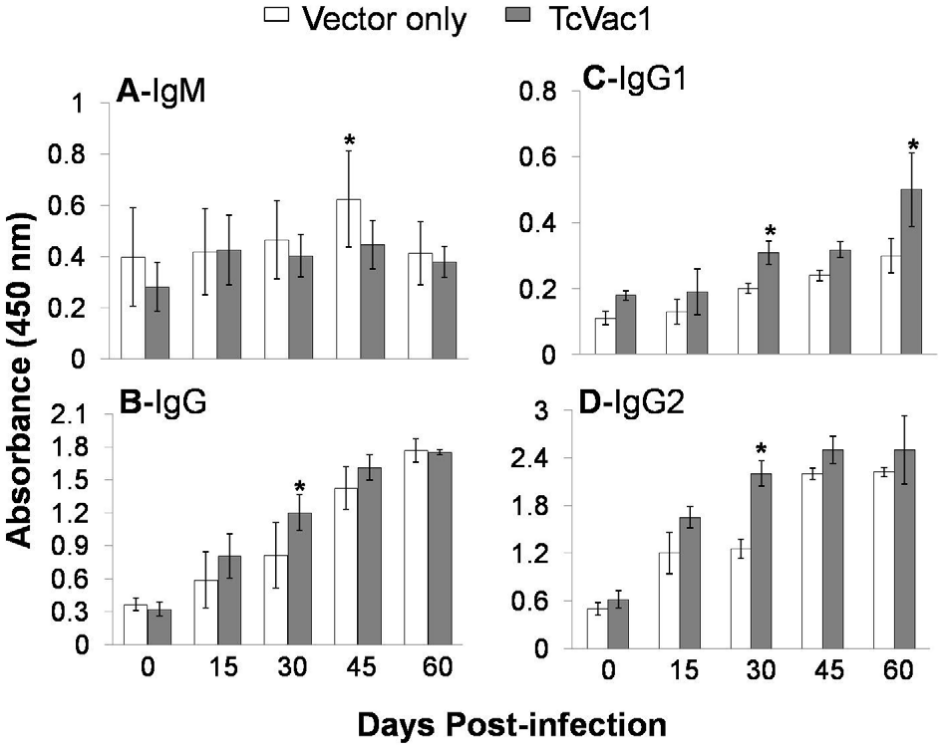
The antibody response to *T. cruzi* infection was polarized to type 1 in vaccinated dogs. Dogs were vaccinated as above, and infected with *T. cruzi*. An ELISA was performed to evaluate sera levels (1∶100-dilution) of *T. cruzi*-specific IgM (**A**), IgG (**B**), IgG1 (**C**), and IgG2 (**D**) antibodies at 0, 15, 30, 45, and 60 days post-infection.

After challenge infection with *T. cruzi*, sera samples were analyzed at 2-week intervals (1∶100 dilution) (Fig. 2). Non-vaccinated/infected dogs exhibited a slight increase in parasite-specific IgM levels (Fig. 2A). All dogs, irrespective of vaccination status, responded to *T. cruzi* infection by a gradual increase in anti-parasite IgG levels (Fig. 2B). The TcVac1-immunized dogs exhibited a faster increase in IgG antibody response to *T. cruzi* infection (Fig. 2B, *p*<0.05) as compared to that detected in non-vaccinated/infected dogs. Likewise, the vaccine-induced dominance of IgG2 antibodies (compared to IgG1 subtype) was significantly expanded after infection (Fig. 2C&D). Together, the results presented in Fig. 1 and Fig. 2 suggested that vaccination of dogs with TcVac1 skewed the antibody response towards Th1 type that was further expanded upon challenge infection with *T. cruzi*.

**Figure 3:**
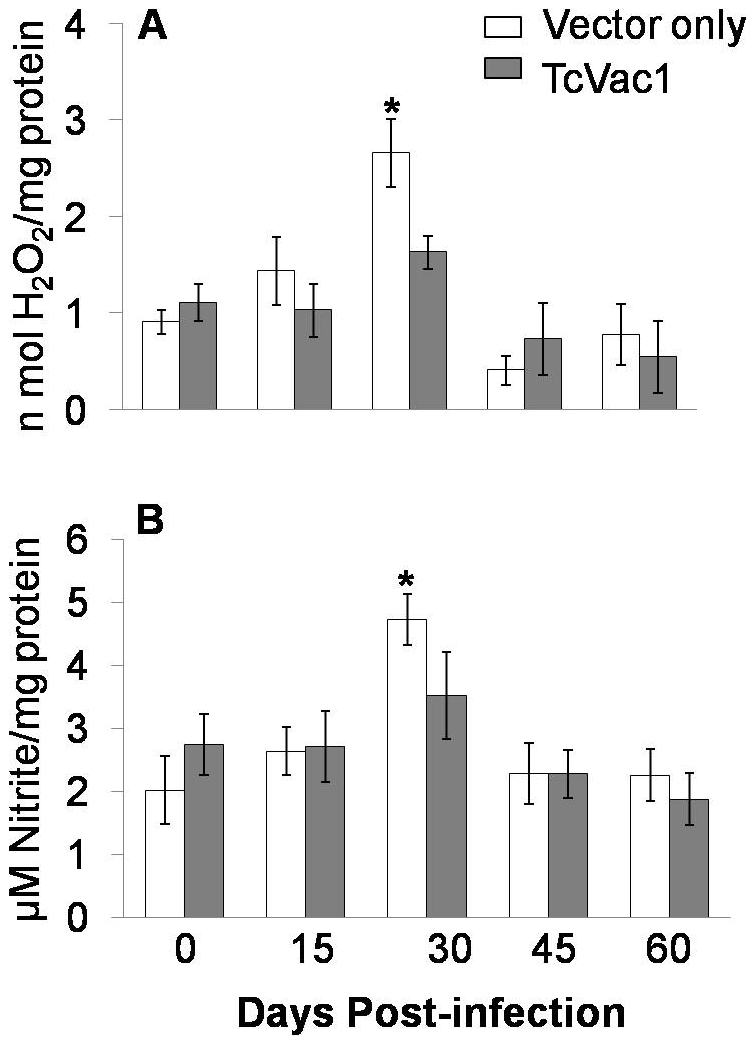
Shown are plasma levels of myeloperoxidase activity (A) and nitrite content (B) in dogs immunized with vector only or TcVac1 vaccine during the course of 0–60 days post-infection. Data are presented as mean ± SD (*p<0.05).

Next, we measured vaccine’s efficacy in activation of phagocytic (neutrophils and macrophages) response to *T. cruzi* by evaluating the plasma level of MPO activity and nitrite contents (Fig. 3). Vaccinated and non-vaccinated dogs exhibited no detectable level of MPO activity before challenge infection (Fig. 3A). After exposure to *T. cruzi*, all dogs responded by a rise in MPO activity. During day 15–30 pi, non-vaccinated/infected and vaccinated/infected dogs exhibited a 2-fold and 25% increase in MPO activity in response to *T. cruzi* infection (Fig. 3A). After day 30 pi, all dogs exhibited a similar decline in circulatory MPO activity. Likewise, the nitrite levels, indicative of iNOS activation and NO production, were increased by 2-fold in non-vaccinated/infected dogs at 30 dpi, while vaccinated/infected dogs exhibited ∼22% increase in plasma nitrite contents (Fig. 3B). These data suggested that immunization with TcVac1 suppressed the *T. cruzi*-mediated activation of phagocytes evidenced by decreased plasma levels of MPO and nitrite in vaccinated/acutely-infected dogs as compared to non-vaccinated controls.

**Figure 4:**
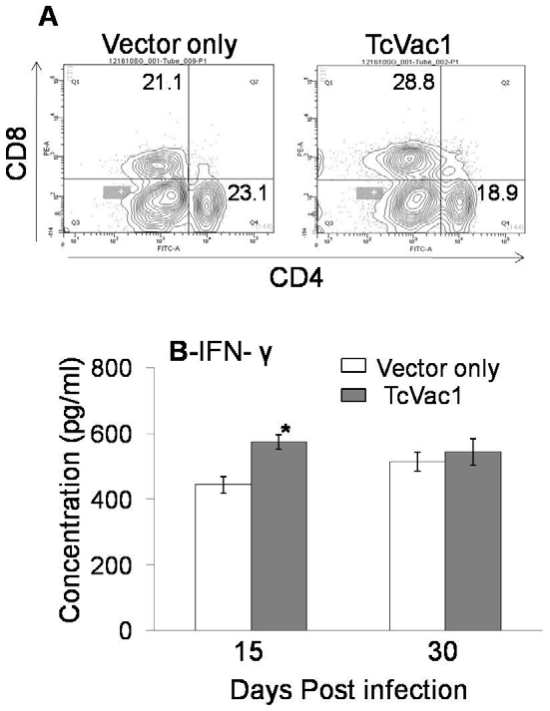
T cell and cytokine response in vaccinated dogs. (**A**) Spleen cells were obtained from vaccinated and non-vaccinated dogs at one year after challenge infection with *T. cruzi*. Spleen cells were incubated for 30 min with FITC-conjugated anti-CD8 and PE-conjugated anti-CD4 antibodies and CD4^+^ and CD8^+^ T cell subsets monitored by flow cytometry. (**B**) The circulatory IFN-γ level was measured by an ELISA. Data are presented as mean ± SD (*p<0.05).

A predominance of CD8^+^ T cells and type 1 cytokines (IFN-γ) is shown to be essential for control of *T. cruzi* infection [32]. All dogs, irrespective of vaccination regimen, responded to *T. cruzi* infection by a strong increase in parasite-specific lymphocyte activation (Fig. 4). The vaccinated/infected dogs exhibited a moderately stronger CD8^+^T cell response as compared to the non-vaccinated/infected dogs that was maintained during acute infection and chronic disease phase (Fig. 4A). The circulatory cytokine levels (IFN-γ and IL-10) were below detection limit before and after immunization with TcVac1. The sera level of IL-10 remained undetectable after challenge infection with *T. cruzi* in all dogs. In comparison, all dogs responded to infection by a rapid increase in circulatory IFN-γ level that was significantly higher in vaccinated/infected dogs as compared to that noted in non-vaccinated/infected dogs (Fig. 4B). These results indicated that TcVac1-immunized dogs were moderately better than non-vaccinated dogs in responding to *T. cruzi* infection by elicitation of higher level of type 1 biased CD8^+^T cell response.

**Figure 5:**
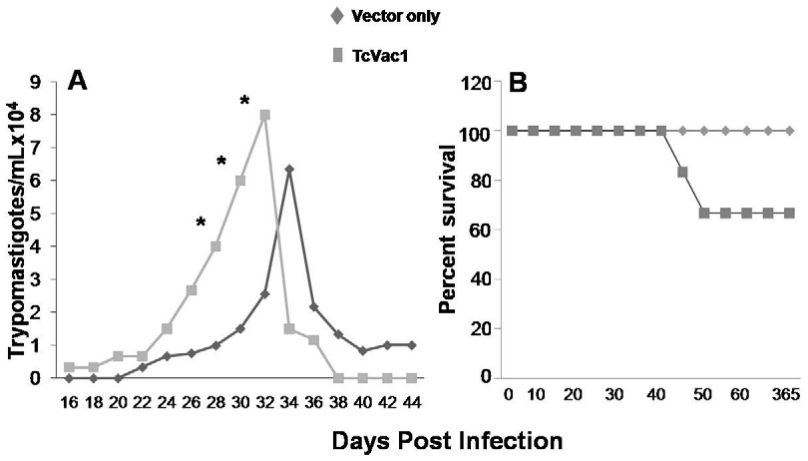
(**A**) Blood parasitemia was determined by light microscopy. Data are presented as mean ± SD (*p<0.05). (**B**) Percent survival from infection.

Detectable parasitemia that peaked during day 30–35 pi was noted in all dogs (Fig. 5A). Dogs vaccinated with TcVac1 exhibited an early rise in parasitemia that was controlled by day 37 pi. In comparison, non-vaccinated/infected dogs exhibited a slight delay in peak parasitemia; however, blood parasitemia persisted beyond day 37 pi. No signs of clinical illness were apparent in vaccinated/infected and non-vaccinated/infected dogs during the physical exam, yet 33% of the TcVac1-vaccinated/infected dogs succumbed during 40–42 dpi (Fig. 5B).

Xenodiagnostic studies were performed to determine if dog’s infectivity to triatomines is altered by vaccination. Triatomines were fed on dogs during acute phase (30 dpi) and after control of acute parasitemia (60 dpi), and feces were analyzed 30 days post-feeding for the detection of parasites by microscopy. In agreement with the peak parasitemia, all triatomines fed at day 30 pi on vaccinated and non-vaccinated dogs became *T. cruzi* positive. Of the 36 triatomines fed on each group of dogs at day 60 pi, 47% (17 out of 36) insects fed on TcVac1-vaccinated/infected dogs and 30% (11 out of 36) insects fed on non-vaccinated/infected dogs died during the incubation period. Of those surviving, we detected *T. cruzi* in feces of 52.63% (10/19) and 84.6% (21/25) of the insects fed on vaccinated/infected and non-vaccinate/infected dogs, respectively. These results indicated that TcVac1 was not effective in preventing infection or early rise in acute parasitemia, and was moderately effective in reducing the time-course of parasitemia and dogs’ infectivity to triatomines after day 37 post-infection.

Normal electrocardiographic readings were noted in all dogs included in the study, before and after immunization. After challenge infection, vaccinated/infected and non-vaccinated/infected dogs exhibited no cardiac alterations up to 30 dpi. By day 60 pi, 67% of non-vaccinated dogs displayed electrocardiographic alterations, including reduced P-R interval, reduced R wave voltage, axis rotated to the right, S-T segment line elevation from the isoelectric line (>0.2 mV), long QT segment, J wave elevation, and sinus tachycardia that were diagnostic of myocarditis, pericarditis, and high degree of myocardiocyte necrosis. Two vaccinated/infected dogs died by day 42 pi due to high electrical conductance problems and arrhythmia. Of remaining, 50% of the vaccinated/infected dogs exhibited at 60 dpi no electrocardiographic alterations, and other 50% exhibited a moderate level of EKG abnormalities including low voltage complex, a positive deviation of S-T with elevation of J-wave, left axel rotation, and tachycardia that were diagnostic of ventricular dilation, myocarditis, and arrhythmia. At one-year post-challenge infection, electrocardiographic analysis revealed a spectrum of cardiac dysfunction in chronically infected dogs. Among the unvaccinated/chronically-infected dogs, 66% exhibited major electrical conduction problems (right axel rotation and LBBB), and 33% exhibited lateral re-polarization problems. Among the vaccinated/chronically-infected dogs, 33% exhibited electrical conduction problems (right axel rotation and LBBB) similar to that noted in unvaccinated/infected dogs, 33% showed minor axel rotation problems, and 33% dogs exhibited normal EKG. On a scale of 0 (normal) to 10 (severe EKG alterations), 66% of non-vaccinated/chronically infected dogs were graded as 10 and 33% as normal (zero EKG alterations). In comparison, 33% of vaccinated/chronically infected dogs were graded normal (0), 33% moderate (score: 5), and 33% with severe electrical conduction problems (score: 10) at one year post-infection.

**Figure 6:**
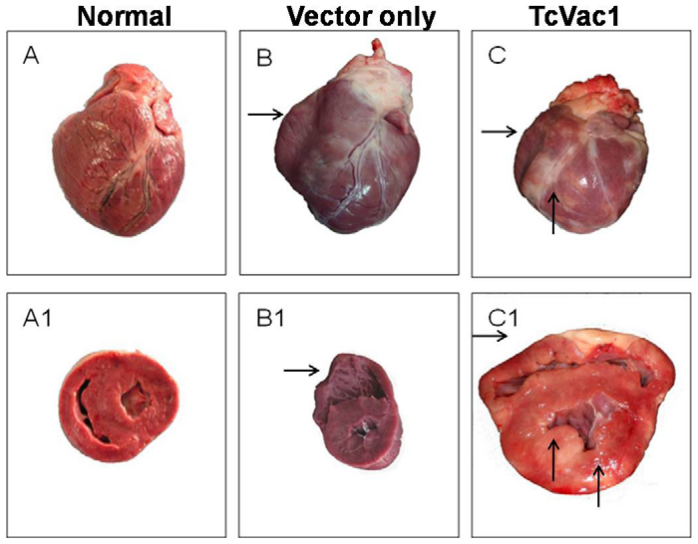
Morphological alterations in the heart. Dogs were vaccinated and infected with *T. cruzi* as above. Shown are representative morphologic alterations of the heart during the acute infection phase (60 dpi) in dogs injected with vector only (**B&B1**) or immunized with TcVac1 (**C&C1**). Images of normal heart (**A&A1**) are shown for comparison. Horizontal arrows show right ventricle wall thinning characteristic of ventricle dilation. Vertical arrows show pale striated epicardium and myocardium, characteristic of necrosis produced after inflammatory response to infection.

Next, we evaluated the pathology of the heart in dogs. Anatomo-pathological analysis of the heart, performed at day 60 pi, showed dilated cardiomyopathy (bi-ventricular dilation) and focal and diffused myocarditis in vaccinated dogs as well as in dogs injected with vector alone (Fig. 6). Irrespective of vaccination status, some animals exhibited whitish zones and rounded edges in the spleen, dilation of esophagus, and pinkish ampoules at the cecum. At one-year post-challenge infection, all dogs had round shaped hearts. Sixty six percent of non-vaccinated/chronically infected dogs exhibited severe right ventricle dilation. In comparison, 66% of vaccinated/chronically-infected dogs exhibited moderate level of right ventricle dilation. Epicardial hemorrhages were seen in 66% dogs from the control group and 33% dogs in the TcVac1 group.

**Figure 7:**
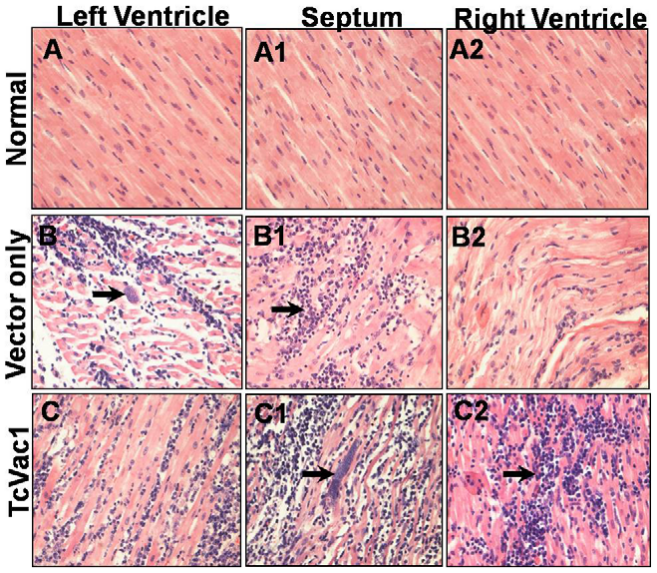
Histological analysis of hearts. Dogs were vaccinated, and challenged with *T. cruzi*. Heart tissue sections (5-ìM) from left ventricle, septum, and right ventricle were obtained at 60 days post-infection (acute phase), and stained with hematoxylin-eosin. Shown are representative micrographs of dogs injected with vector only (**B,B1,B2**) or immunized with TcVac1 (**C,C1,C2**). Micrographs from normal/uninfected dogs (**A,A1,A2**) are shown for comparison. Vertical arrows show amastigote nests and horizontal arrows show lymphocyte infiltration, and cardiomyocytes destruction.

Histopathology studies on day 60 pi demonstrated some differences between two groups (Fig. 7). In epicardium of dogs vaccinated with TcVac1, non-suppurative moderate to severe myocarditis with focal or zonal mononuclear and polymorphonuclear inflammatory infiltrate associated with presence of amastigotes nests and severe active necrosis was generally noted. In non-vaccinated/infected dogs, histopathological findings were similar to that noted in vaccinated dogs with the exception that inflammatory infiltrate tended to be mainly constituted of mononuclear rather than polymorphonuclear cell type (Fig. 7). In the myocardium, vaccinated and non-vaccinated dogs exhibited multiple coagulative necrosis foci with mononuclear infiltration and some necrotic areas with polymorphonuclear and neutrophil infiltration. The diffused multi-focal and zonal mononuclear inflammatory infiltrate and hemorrhagic areas in myocardium appeared to be larger in vaccinated/infected dogs (Fig. 7C, C2) as compared to non-vaccinated/infected dogs (Fig. 7B, B2). Vaccinated/infected dogs also showed abundant cellular detritus in myocardium. Mononuclear infiltration was moderate in ventricles and septum of all dogs. Folding of myocardial fibers in right ventricle was observed in both vaccinated/acutely-infected and non-vaccinated/acutely-infected dogs. Mural multifocal endocarditis with mononuclear infiltration was also noted in dogs from the two groups. Abundant amastigote nests (range 18–21 per microscopic field (mf)) were found in each region (right and left ventricles, and septum) of the heart of TcVac1-vaccinated/acutely-infected dogs. Non-vaccinated/infected dogs exhibited, in general, lesser number of parasite foci in equivalent studied areas. Statistical analysis showed that overall, the number of myocardial necrotic foci, lymphocyte infiltration foci and number of amastigote nests were more abundant in TcVac1-vaccinated dogs than was observed in non-vaccinated/infected dogs at 60 dpi (p<0.05) (Fig. 7).

At one-year post-challenge infection, severe myocardial inflammation persisted in 66% of the non-vaccinated/infected dogs while remaining 33% exhibited slight inflammatory infiltrate in the heart. In comparison, 66% of vaccinated/chronically infected dogs exhibited moderate level of myocardial inflammatory infiltrate. Slight to moderate presence of connective tissue was apparent in all chronically infected dogs; however, it was more evident in TcVac1-vaccinated/chronic dogs. Folding of myocardial fibers and vacuolization of Purkinje fibers was observed in 33% of vaccinated/chronically infected dogs.

## Discussion

The objective of the present study was to test the efficacy of a multi-component DNA vaccine (TcVac1) in dogs. The antigenic candidates included in TcVac1 were identified by computational analysis of *T. cruzi* sequence database and selected because they were conserved among several clinically relevant *T. cruzi* strains, expressed in infective and intracellular stages of *T. cruzi* [9], and recognized by the antibody and T cell response in infected mice [32] and humans (unpublished data). When delivered as a DNA vaccine in mice, TcG1, TcG2 and TcG4 elicited trypanolytic antibody response and Th1 cytokines (e.g. IFN-γ) [9] that resulted in significant protection from acute infection and chronic disease severity. We utilized IL-12 and GM-CSF expression plasmids as adjuvants as these cytokines induce type 1 B and T cell responses [33], and shown to significantly enhance the protective immunity elicited by the vaccine candidates in mice [8], [39] and dogs [33]. To the best of our knowledge, this is the first report testing the prophylactic and transmission-blocking efficacy of DNA vaccine against *T. cruzi* in dogs.

Immunization of dogs with TcVac1 resulted in elicitation of antigen-specific and parasite-specific antibody response that was dominated by IgG2 subtype. The delivery of booster doses of vaccine resulted in no significant increase in antibody response that could be explained, at least partially, by the fact that DNA delivery system, used in this study, is known to drive a low level of antigen expression. Several investigators have reported that needle delivery of DNA vaccines in muscle induce low immune response in large animals and humans, even when 1000-fold higher doses of DNA than those proved to be effective in rodents were given [40], [41]. Other DNA vaccine delivery systems such as gene gun (biolistic gun) [42], [43], adenovirus or vaccinia virus delivery vectors [44], replicating attenuated strains of intracellular microorganisms, such as *Salmonella* [45] have shown promising results in eliciting antigen expression. Additionally, heterologous prime/boost approaches are noted to be more effective in eliciting stronger, long-term immunity against intracellular pathogens [46], [47], to be tested in future studies.

Despite low vaccine-induced antibody response, vaccinated dogs, upon challenge infection with *T. cruzi*, exhibited an early expansion in antibody response. The Ig (G+M) response during acute phase was of higher magnitude in vaccinated/infected dogs than that observed in dogs injected with vector alone. The IgG response in vaccinated/infected dogs was primarily of the Th1 type with IgG2/IgG1 ratios being >1, known to provide protection from acute infection in dogs [48], [49]. The higher level and rapid expansion of antibody titers indicates that TcVac1 primed the B cell response that was expanded upon exposure to *T. cruzi*. Previously, we have shown that TcG1-, TcG2- and TcG4*-*specific antibodies, elicited in vaccinated mice, were lytic in nature, and efficiently killed trypomastigotes in a complement-dependent manner [9]. In this study, our observation of a shorter detectable parasitemic period of 37 days in vaccinated/infected dogs than that noted in control dogs (44 days) indicate that antibody response primed by vaccination with TcVac1 was lytic in nature and contributed to a control of blood parasitemia. Yet, immunization with TcVac1 failed to prevent peak parasitemia. This was likely because other components of immune system, i.e., innate response constituted by phagocytes and type 1 biased CD8^+^ T cell response, were not strongly primed by vaccine or expanded upon challenge infection in vaccinated dogs. It is well documented that phagocytes, through activation of NADPH oxidase, MPO, and iNOS activities and production of cytotoxic reactive oxygen and nitrogen species, play an important role in control of *T. cruzi* [37], [50]-[52]. Numerous studies have also demonstrated that an efficient control of acute parasitemia requires concerted activities of Th1 helper cells, and cytotoxic CD8^+^ T lymphocytes (CTLs) (reviewed in [53], [54]). Vaccination with TcVac1 resulted in a suppression of phagocytes’ response to challenge infection as was evidenced by decreased activation of MPO and iNOS activity in vaccinated/infected dogs as compared to that noted in non-vaccinated/acutely infected dogs. Equally, immunization with TcVac1 resulted in a significant but only moderately better expansion of CD8^+^ T cells and IFN-γ levels upon challenge infection when compared to that noted in non-vaccinated/infected dogs. Consequently, it was not surprising to find no significant decline in infectivity of vaccinated dogs to triatomines during the acute period of infection. All triatomines, fed on vaccinated/infected or non-vaccinated/infected dogs when dogs were exhibiting peak parasitemia (day 30 pi), and analyzed at day 60 post-feeding, were infected evidenced by fecal presence of *T. cruzi*. However, at 60 dpi, vaccinated dogs exhibited a better control of parasitemia and moderately reduced infectivity to triatomines (52.63% versus 84.6% infected). The mathematical modeling of transmission dynamics [55] and other studies using insecticide-treated dog collars [56], [57] indicate that a decline in infectivity to <20% would be required to block vectorial transmission of *T. cruzi* to humans. Thus, we surmise that current formulation of TcVac1, though provided a decline in dogs’ infectivity to triatomines after peak parasitemia, would not be effective in blocking the transmission cycle, and further improvement in vaccination strategy is required.

Infection of dogs with SylvioX10/4 strain of *T. cruzi* produced reproducible acute phase and chronic pathology as we have previously reported [18], causing sudden death in some of the infected dogs, and cardiomyopathic changes in most of the infected animals during acute stage. EKG alterations were found in more than half of the acutely infected dogs and ranged from electrical conduction problems, ventricular dilatations, pericarditis, myocarditis, high lateral necrosis, and arrhythmia. Most of these changes were validated by necropsy and histopathology findings, thus, confirming that this *T. cruzi* strain is highly pathogenic in dogs. Despite a similar or higher infiltration of inflammatory infiltrate in the heart, vaccinated dogs exhibited a significantly higher number of amastigote nests (P<0.05) in cardiac tissue than was observed in non-vaccinated control dogs (Fig. 7). Others have reported a direct correlation between *in vitro* infectivity and blood parasitism kinetics with heart parasitism intensity during long-term infection of Beagle dogs [58], [59]. Because of high inflammatory infiltrate and tissue parasite burden, two of the vaccinated dogs exhibited myocarditis and died suddenly due to arrhythmia.

Chronically infected/vaccinated dogs were better equipped in controlling the disease symptoms. EKG findings demonstrated mild-to-moderate cardiac alterations in animals given TcVac1 vaccine while severe EKG alterations persisted in dogs injected with vector alone. These findings were supported by anatomo-pathological analysis performed at one-year post-challenge infection. Anatomo-pathological lesions and epicardial hemorrhages were fewer and moderate in TcVac1-vaccinated/chronically infected dogs as compared to non-vaccinated/chronic dogs that exhibited severe right ventricle dilation and extensive epicardial hemorrhages. These findings were observed despite no decline in inflammatory infiltrate in the heart in chronically infected dogs. These data indicate that TcVac1-induced immunity was at least partially effective in controlling the clinical progression of cardiac disease severity in chagasic dogs.

Summarizing, in this study, we tested a multi-component DNA vaccine against *T. cruzi* infection in dogs. Our data showed that TcVac1 geared a modest parasite- and antigen-specific type 1 antibody and CD8^+^ T cell response that was effective in providing an early control of acute parasitemia and moderately decreased the infectivity of dogs to triatomines. However, tissue parasite burden was not controlled in vaccinated dogs, likely due to suppression of phagocytic cell response, evidenced by decreased myeloperoxidase and nitrite (iNOS) levels in immunized dogs. Despite this, vaccinated dogs exhibited a moderate decline in cardiac alterations determined by EKG and anatomo-/histo-pathological analysis during chronic stage of disease development. Overall, our data demonstrated that TcVac1-elicited immunity provided a partial protection from chronic Chagas disease and provided an impetus to further improve the vaccination strategy against Chagas disease.

## References

[1] World Health Organization (2010). http://apps.who.int/gb/ebwha/pdf_files/WHA63/A63_17-en.pdf.

[2] Coura JR, Dias JC (2009). Epidemiology, control, and surveillance of Chagas disease: 100
years after its discovery.. Mem Inst Oswaldo Cruz.

[3] Rassi A, Rassi A, Marin-Neto JA (2010). Chagas disease.. Lancet.

[4] Silveira EL, Claser C, Haolla FA, Zanella LG, Rodrigues MM (2008). Novel protective antigens expressed by *Trypanosoma
cruzi* amastigotes provide immunity to mice highly susceptible
to Chagas' disease.. Clin Vaccine Immunol.

[5] de Alencar BC, Persechini PM, Haolla FA, de Oliveira G, Silverio JC (2009). Perforin and gamma interferon expression are required for
CD4^+^ and CD8^+^ T-cell-dependent
protective immunity against a human parasite, *Trypanosoma
cruzi*, elicited by heterologous plasmid DNA prime-recombinant
adenovirus 5 boost vaccination.. Infect Immun.

[6] Bhatia V, Garg N (2005). Current status and future prospects for a vaccine against
American trypanosomiasis.. Expert Rev Vaccines.

[7] Vázquez-Chagoyán JC, Gupta S, Garg NJ (2011). Vaccine development against *Trypanosoma cruzi*
and Chagas disease..

[8] Garg N, Tarleton RL (2002). Genetic immunization elicits antigen-specific protective immune
responses and decreases disease severity in *Trypanosoma
cruzi* infection.. Infect Immun.

[9] Bhatia V, Sinha M, Luxon B, Garg N (2004). Utility of *Trypanosoma cruzi* sequence database
for the identification of potential vaccine candidates: *In
silico* and *in vitro* screening.. Infect Immun.

[10] Bhatia V, Garg NJ (2008). Previously unrecognized vaccine candidates control
*Trypanosoma cruzi* infection and immunopathology in
mice.. Clin Vaccine Immunol.

[11] de Lana M, Chiari E, Tafuri WL (1992). Experimental Chagas' disease in dogs.. Mem Inst Oswaldo Cruz.

[12] Tafuri WL, de Lana M, Chiari E, Caliari MV, Bambirra EA (1988). Dogs as experimental models for the study of the natural course
of Chagas disease.. Rev Soc Bras Med Trop.

[13] Andrade ZA, Andrade SG, Sadigursky M, Wenthold RJ, Hilbert SL (1997). The indeterminate phase of Chagas' disease: ultrastructural
characterization of cardiac changes in the canine model.. Am J Trop Med Hyg.

[14] Guedes PM, Veloso VM, Afonso LC, Caliari MV, Carneiro CM (2009). Development of chronic cardiomyopathy in canine Chagas disease
correlates with high IFN-gamma, TNF-alpha, and low IL-10 production during
the acute infection phase.. Vet Immunol Immunopathol.

[15] Barbabosa-Pliego A, Gil PC, Hernandez DO, Apparicio-Burgos E, de Oca-Kimenez RM (2010). Prevalence of *Trypanosoma cruzi* in dogs (Canis
familiaris) and triatomines during 2008 in a sanitary region of the State of
Mexico, Mexico.. Vector Borne Zoonotic Dis.

[16] Carneiro CM, Martins-Filho OA, Reis AB, Veloso VM, Araujo FM (2007). Differential impact of metacyclic and blood trypomastigotes on
parasitological, serological and phenotypic features triggered during acute
*Trypanosoma cruzi* infection in dogs.. Acta Trop.

[17] Andrade ZA (1991). Pathogenesis of Chagas disease.. Res Immunol.

[18] Barbabosa-Pliego A, Díaz-Albiter HM, Ochoa-Garcia L, Aparicio-Burgos E, López-Heydeck S (2009). *Trypanosoma cruzi* circulating in the southern
region of the State of Mexico (Zumpahuacan) is pathogenic: a dog
model.. Am J Trop Med Hyg.

[19] Goble FC (1952). Observations on experimental Chagas' disease in
dogs.. Am J Trop Med Hyg.

[20] Lopes ER, Chapadeiro E, Andrade ZA, Almeida HO, Rocha A (1981). Pathological anatomy of hearts from asymptomatic Chagas disease
patients dying in a violent manner.. Mem Inst Oswaldo Cruz.

[21] Cruz-Chan JV, Bolio-Gonzalez M, Colin-Flores R, Ramirez-Sierra MJ, Quijano-Hernandez I (2009). Immunopathology of natural infection with *Trypanosoma
cruzi* in dogs.. Vet Parasitol.

[22] Laranja FS, Dias E, Duarte E, Pellegrino J (1951). Clinical and epidemiological observations on Chagas' disease
in western Minas Gerais.. Hospital (Rio J).

[23] Laranja FS, Andrade ZA (1980). Chronic cardiac form of Chagas disease in dogs.. Arq Bras Cardiol.

[24] Gurtler RE, Kravetz FO, Petersen RM, Lauricella MA, Wisnivesky-Colli C (1990). The prevalence of *Trypanosoma cruzi* and the
demography of dog populations after insecticidal spraying of houses: a
predictive model.. Ann Trop Med Parasitol.

[25] Gurtler RE, Cecere MC, Castanera MB, Canale D, Lauricella MA (1996). Probability of infection with *Trypanosoma cruzi*
of the vector *Triatoma infestans* fed on infected humans and
dogs in northwest Argentina.. Am J Trop Med Hyg.

[26] Gurtler RE, Cecere MC, Lauricella MA, Cardinal MV, Kitron U (2007). Domestic dogs and cats as sources of *Trypanosoma
cruzi* infection in rural northwestern
Argentina.. Parasitology.

[27] Meurs KM, Anthony MA, Slater M, Miller MW (1998). Chronic *Trypanosoma cruzi* infection in dogs: 11
cases (1987-1996).. J Am Vet Med Assoc.

[28] Barr SC, Van Beek O, Carlisle-Nowak MS, Lopez JW, Kirchhoff LV (1995). *Trypanosoma cruzi* infection in Walker hounds
from Virginia.. Am J Vet Res.

[29] Gurtler RE, Cohen JE, Cecere MC, Chuit R (1997). Shifting host choices of the vector of Chagas disease
*Triatoma infecstans* and the availability of hosts in
houses in north-west Argentina.. J Appl Ecol.

[30] NOM-033-ZOO NOM (1995). Sacrificio Humanitario de los Animales Domésticos y
Silvestre,. http://www.cuautitlan.unam.mx/descargas/cicuae/normas/Norma033.pdf.

[31] NOM-062-ZOO NOM (1999). Especificaciones Técnicas para la Producción,
Cuidado y Uso de los Animales del Laboratorio,. http://www.fmvz.unam.mx/fmvz/principal/archivos/062ZOO.pdf.

[32] Gupta S, Garg NJ (2010). Prophylactic efficacy of TcVac2 against *Trypanosoma
cruzi* in mice.. PLoS Negl Trop Dis.

[33] Saldarriaga OA, Perez LE, Travi BL, Melby PC (2006). Selective enhancement of the type 1 cytokine response by
expression of a canine interleukin (IL)-12 fused heterodimeric
DNA.. Vet Immunol Immunopathol.

[34] Portela-Lindoso AA, Shikanai-Yasuda MA (2003). Chronic Chagas' disease: from xenodiagnosis and hemoculture
to polymerase chain reaction.. Rev Saude Publica.

[35] Basso B, Castro I, Introini V, Gil P, Truyens C, Moretti E (2007). Vaccination with *Trypanosoma rangeli* reduces the
infectiousness of dogs experimentally infected with *Trypanosoma
cruzi.*. Vaccine.

[36] Bradley PP, Priebat DA, Christensen RD, Rothstein G (1982). Measurement of cutaneous inflammation: estimation of neutrophil
content with an enzyme marker.. J Invest Dermatol.

[37] Dhiman M, Estrada-Franco JG, Pando J, Ramirez-Aguilar F, Spratt H (2009). Increased myeloperoxidase activity and protein nitration are
indicators of inflammation in chagasic patients.. Clinical and Vaccine Immunology.

[38] Garg N, Popov VL, Papaconstantinou J (2003). Profiling gene transcription reveals a deficiency of
mitochondrial oxidative phosphorylation in *Trypanosoma
cruzi*-infected murine hearts: implications in chagasic
myocarditis development.. Biochim Biophys Acta.

[39] Garg N, Tarleton RL (1998). Elicitation of protective cellular and humoral immune responses
to *Trypanosoma cruzi* infection using DNA vaccines can be
augmented with cytokines..

[40] Donnelly JJ, Wahren B, Liu MA (2005). DNA vaccines: progress and challenges.. J Immunol.

[41] Endmann A, Baden M, Weisermann E, Kapp K, Schroff M (2010). Immune response induced by a linear DNA vector: influence of
dose, formulation and route of injection.. Vaccine.

[42] Sakai T, Hisaeda H, Nakano Y, Ishikawa H, Maekawa Y (2000). Gene gun-mediated delivery of an interleukin-12 expression
plasmid protects against infections with the intracellular protozoan
parasites *Leishmania major* and *Trypanosoma
cruzi* in mice.. Immunology.

[43] Weinberg M, Waterman S, Lucas CA, Falcon VC, Morales PK (2003). The U.S.-Mexico border infectious disease surveillance project:
establishing bi-national border surveillance.. Emerg Infect Dis.

[44] Miyahira Y, Takashima Y, Kobayashi S, Matsumoto Y, Takeuchi T (2005). Immune responses against a single CD8^+^ T-cell
epitope induced by virus vector vaccination can successfully control
*Trypanosoma cruzi* infection.. Infect Immun.

[45] Cazorla SI, Becker PD, Frank FM, Ebensen T, Sartori MJ (2008). Oral vaccination with *Salmonella enterica* as a
cruzipain-DNA delivery system confers protective immunity against
*Trypanosoma cruzi*.. Infect Immun.

[46] Moore AC, Hill AV (2004). Progress in DNA-based heterologous prime-boost immunization
strategies for malaria.. Immunol Rev.

[47] Gilbert SC, Moorthy VS, Andrews L, Pathan AA, McConkey SJ (2006). Synergistic DNA-MVA prime-boost vaccination regimes for malaria
and tuberculosis.. Vaccine.

[48] Guedes PM, Veloso VM, Gollob KJ, Afonso LC, Caldas IS (2008). IgG isotype profile is correlated with cardiomegaly in beagle
dogs infected with distinct *Trypanosoma cruzi*
strains.. Vet Immunol Immunopathol.

[49] Coura-Vital W, Carneiro CM, Martins HR, de Lana M, Veloso VM (2008). *Trypanosoma cruzi*: immunoglobulin isotype
profiles during the acute phase of canine experimental infection with
metacyclic or blood trypomastigotes.. Exp Parasitol.

[50] Cardoni RL, Antunez MI, Morales C, Nantes IR (1997). Release of reactive oxygen species by phagocytic cells in
response to live parasites in mice infected with *Trypanosoma
cruzi*.. Am J Trop Med Hyg.

[51] Piacenza L, Alvarez MN, Peluffo G, Radi R (2009). Fighting the oxidative assault: the *Trypanosoma
cruzi* journey to infection.. Curr Opin Microbiol.

[52] Alvarez MN, Piacenza L, Irigoin F, Peluffo G, Radi R (2004). Macrophage-derived peroxynitrite diffusion and toxicity to
*Trypanosoma cruzi*.. Arch Biochem Biophys.

[53] Zacks MA, Wen JJ, Vyatkina G, Bhatia V, Garg N (2005). An overview of chagasic cardiomyopathy: pathogenic importance of
oxidative stress.. An Acad Bras Cienc.

[54] Junqueira C, Caetano B, Bartholomeu DC, Melo MB, Ropert C (2010). The endless race between *Trypanosoma cruzi* and
host immunity: lessons for and beyond Chagas disease.. Expert Rev Mol Med.

[55] Cohen JE, Gurtler RE (2001). Modeling household transmission of American
trypanosomiasis.. Science.

[56] Reithinger R, Ceballos L, Stariolo R, Davies CR, Gurtler RE (2005). Chagas disease control: deltamethrin-treated collars reduce
*Triatoma infestans* feeding success on
dogs.. Trans R Soc Trop Med Hyg.

[57] Reithinger R, Ceballos L, Stariolo R, Davies CR, Gurtler RE (2006). Extinction of experimental *Triatoma infestans*
populations following continuous exposure to dogs wearing
deltamethrin-treated collars.. Am J Trop Med Hyg.

[58] Guedes PM, Veloso VM, Caliari MV, Carneiro CM, Souza SM (2007). *Trypanosoma cruzi* high infectivity in vitro is
related to cardiac lesions during long-term infection in beagle
dogs.. Mem Inst Oswaldo Cruz.

[59] Veloso VM, Guedes PM, Andrade IM, Caldas IS, Martins HR (2008). *Trypanosoma cruzi*: blood parasitism kinetics and
their correlation with heart parasitism intensity during long-term infection
of beagle dogs.. Mem Inst Oswaldo Cruz.

